# Cost-effectiveness of Antivenoms for Snakebite Envenoming in Nigeria

**DOI:** 10.1371/journal.pntd.0003381

**Published:** 2015-01-08

**Authors:** Abdulrazaq G. Habib, Mohammed Lamorde, Mahmood M. Dalhat, Zaiyad G. Habib, Andreas Kuznik

**Affiliations:** 1 Infectious & Tropical Diseases Unit, College of Health Sciences, Bayero University Kano, Nigeria; 2 Infectious Diseases Institute, Makerere College of Health Sciences, Kampala, Uganda; Liverpool School of Tropical Medicine, United Kingdom

## Abstract

**Background:**

Snakebite envenoming is a major public health problem throughout the rural tropics. Antivenom is effective in reducing mortality and remains the mainstay of therapy. This study aimed to determine the cost-effectiveness of using effective antivenoms for Snakebite envenoming in Nigeria.

**Methodology:**

Economic analysis was conducted from a public healthcare system perspective. Estimates of model inputs were obtained from the literature. Incremental Cost Effectiveness Ratios (ICERs) were quantified as deaths and Disability-Adjusted-Life-Years (DALY) averted from antivenom therapy. A decision analytic model was developed and analyzed with the following model base-case parameter estimates: type of snakes causing bites, antivenom effectiveness to prevent death, untreated mortality, risk of Early Adverse Reactions (EAR), mortality risk from EAR, mean age at bite and remaining life expectancy, and disability risk (amputation). End-user costs applied included: costs of diagnosing and monitoring envenoming, antivenom drug cost, supportive care, shipping/freezing antivenom, transportation to-and-from hospital and feeding costs while on admission, management of antivenom EAR and free alternative snakebite care for ineffective antivenom.

**Principal Findings:**

We calculated a cost/death averted of ($2330.16) and cost/DALY averted of $99.61 discounted and $56.88 undiscounted. Varying antivenom effectiveness through the 95% confidence interval from 55% to 86% yield a cost/DALY averted of $137.02 to $86.61 respectively. Similarly, varying the prevalence of envenoming caused by carpet viper from 0% to 96% yield a cost/DALY averted of $254.18 to $78.25 respectively. More effective antivenoms and carpet viper envenoming rather than non-carpet viper envenoming were associated with lower cost/DALY averted.

**Conclusions/Significance:**

Treatment of snakebite envenoming in Nigeria is cost-effective with a cost/death averted of $2330.16 and cost/DALY averted of $99.61 discounted, lower than the country's gross domestic product per capita of $1555 (2013). Expanding access to effective antivenoms to larger segments of the Nigerian population should be a considered a priority.

## Author Summary

Snake bite is a major public health problem throughout rural communities in West Africa and leads to a significant number of deaths and disabilities per year. Even though effective antivenoms exist against the locally prevalent carpet viper and other poisonous snakes, they are generally not available in community settings, possibly because of their high acquisition cost. We evaluated the cost-effectiveness of making antivenom more broadly available in Nigeria by comparing the treatment costs associated with antivenom therapy against their medical benefit in reducing the risk of mortality. We find that the incremental cost effectiveness ratio (ICER) associated with making antivenom available in Nigeria was $2,330 per death averted and $100 per disability adjusted life year (DALY) averted. Both of these suggest that snakebite antivenom is highly cost-effective in Nigeria and they also compare very favorably against other commonly funded health interventions for which similar estimates exist. Since a substantial reduction in mortality and DALYs could be achieved at a relatively modest upfront cost, expanding access to antivenom to broader parts of the population should be a priority consideration for future investments in healthcare.

## Introduction

Snakebite envenoming is a major public health problem among agricultural communities in the savanna region of West Africa [1]–[3]. A recent global appraisal estimated an incidence of envenomings in West Africa of 8.87–93.3/100,000 persons per year (PPY) and a mortality rate of 0.504–5.9/100,000 PPY [4]. Another recent study estimated snakebite incidence of 54/100,000 PPY and a mortality of 1.35/100,000 PPY occurring annually in West Africa [3]. However, aggregate estimates for West Africa do not fully reflect the substantial regional variability in snakebite incidence. For example, estimates from parts of the Benue valley in northeastern Nigeria reported an incidence as high as of 497 per 100,000 PPY [5], nearly 10-fold the regional average.

Most commonly, snakebite envenoming in Nigeria results from carpet viper (*Echis ocellatus*) attacks, which accounts for at least 66% of all snakebites. However its range is primarily limited to the savannah regions of Nigeria [1], [6], [7], [8], [9]. Carpet viper envenoming presents with swelling of the bitten limb and a clotting disorder (incoagulable blood in the 20 minutes Whole Blood Clotting Test, 20WBCT) that manifests as local and/or systemic bleeding. Resulting anaemia and shock may ultimately lead to death [1], [6], [7]. Non-clotting blood in the 20WBCT is diagnostic of carpet viper envenoming and is used to guide and monitor response to antivenom therapy [1], [6], [7]. In Nigeria non-carpet viper envenoming mainly results from African spitting cobra (*Naja nigricollis*), puff-adder (*Bitis arietans*), mamba (*Dendroaspis polylepis*), burrowing asp or stiletto snake (*Atractaspis microlepidota*), night adder (*Causus maculatus*) and very rarely boomslang (*Dispholidus typus*). With the exception of boomslang envenoming from them present with negative or normal clotting on 20WBCT, local swelling, necrosis and tissue reaction especially following puff adder, and paralysis particularly from Egyptian or forest cobra and mamba bites [10]–[13]. The mortality rate from non-carpet viper envenoming is generally lower [5], [10], [11], [12], [14], [15], but snakebites may lead to blindness, malignant ulcers, pregnancy loss, physical and psychological impairment, scarring, permanent residual disability and loss of productivity following hospitalization and incapacitation [3], [11], [14]–[19].

Clinical response to effective antivenom for carpet viper envenoming is often rapid with restoration of blood coagulability and resolution of spontaneous haemorrhage. Snakebite antivenom is effective in reducing the risk of mortality and remains the mainstay of therapy against carpet viper envenoming [1], [7], [20]. Nevertheless, administration of antivenom carries the risk of early adverse reactions (EAR) which may, in rare cases, lead to death [21], [22]. These EAR may require specific treatment and pre-medication given prior to antivenom administration may reduce risk of EAR occurrence [22], [23], [24]. Antivenoms are usually liquid formulations that require refrigerated transportation, and they have a shelf life of approximately 3 years [25], [26]. The average cost per dose is US$124 (range US$55–$640) depending on the manufacturer [27]. In settings where the cost per dose for treatments of other diseases of public health significance could be lower, assessing the health economic value of antivenoms may be of interest to policy makers. However, few economic evaluations have been conducted, and they either concentrated on cost of production of antivenom or were of a preliminary nature on cost per Disability Adjusted Life Year (DALY) averted following carpet viper envenoming [28], [29]. Here, we evaluated cost-effectiveness of using antivenom in Nigeria to manage snakebite envenoming by calculating incremental cost-effectiveness ratios (ICERs) of cost per death averted and cost per DALY averted. The analysis was conducted from the perspective of healthcare system to aid policy makers in evaluating whether or not to make antivenoms more widely available.

## Methods

### Model Overview

**Figure 1:**
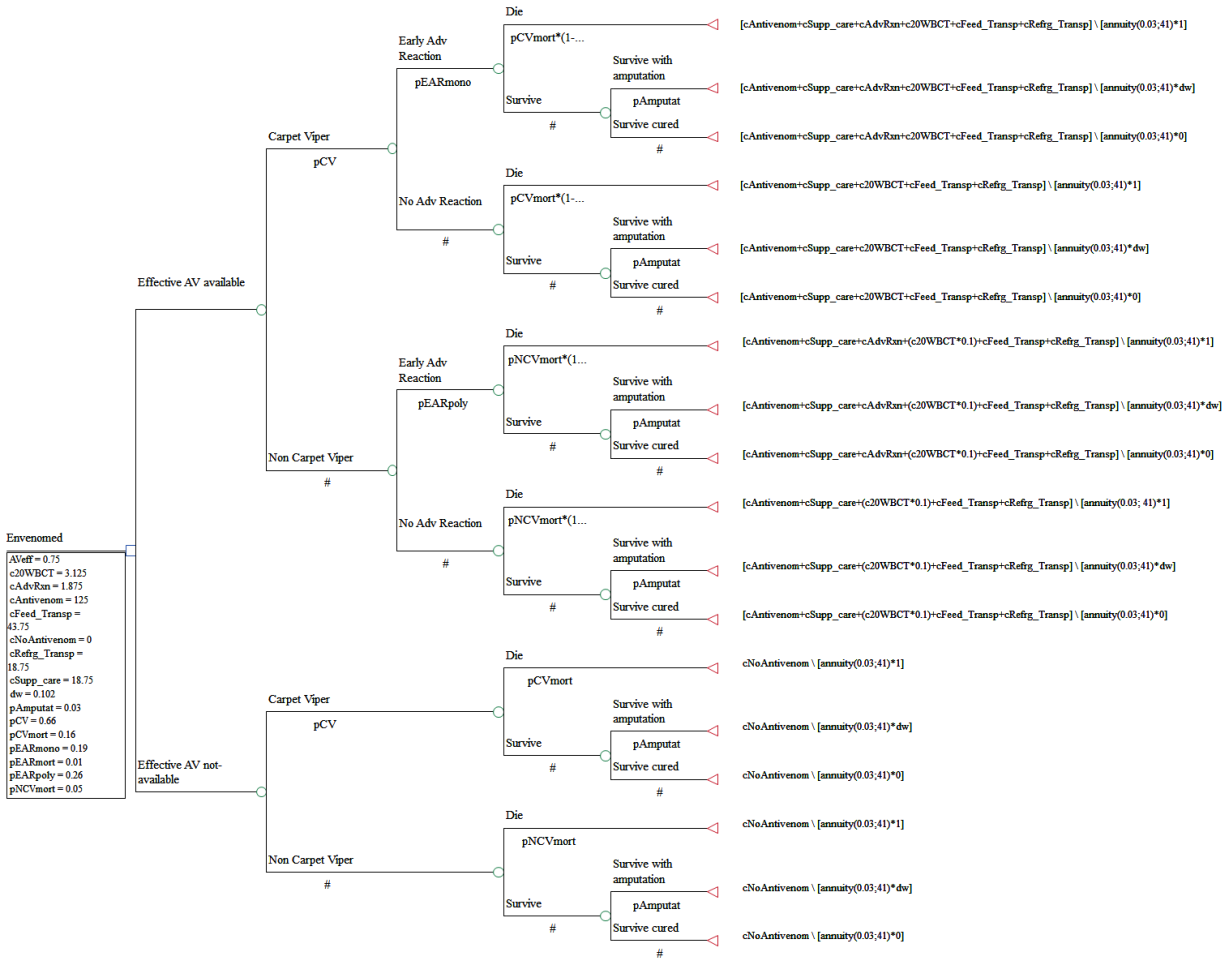
Decision tree for managing snakebite envenoming with or without antivenom. **Model Parameter Definitions**: c20WBCTest  =  cost of 20 minutes Whole Blood Clotting Test on 10 occassions over 7 days at diagnoses and monitoring; cAdvReaction  =  Cost of managing early adverse reactions; cAntivenom  =  Cost of Antivenom; cFeed_Transp  =  Cost of transporation and stay in Hospital for 7 days; cRefrg_Transp  =  Cost of shipping and refrigeration; cNoAntivenom  =  Cost of management without effective antivenoms either traditional/herbal care or other alternatives; cSupp_care  =  Cost of supportive care. All costs are in Nigerian Naira. antivenomeff  =  Effectiveness of antivenom to prevent death; pEARmono  =  probability of early adverse reactionswith monospecific antivenom; pEARpoly  =  probability of early adverse reactionswith polyspecific antivenom; pEARmort  =  probability of dying following effective antivenom and early adverse reactions; pCVmort  =  probability of dying following carpet viper envenoming; pNCVmort  =  probability of dying following non-carpet viper envenoming; pCV  =  proportion of envenoming due to carpet viper; pDisabl  =  probability of disability; dw  =  disability weighting of consequences of snakebite envenoming; x =  effect of adrenaline premedication reduction of risk of early adverse reactions.

A decision analytic model (Fig. 1) was developed to estimate health outcomes and costs associated with the availability and use of geographically appropriate and effective antivenoms for snakebite envenoming in Nigeria. The model was restricted to only envenomed victims or about 40% of bites [15], [29]. Snakebites not leading to morbidity (e.g., dry bites) or bites from non-venomous snakes were excluded. Only 2.5% of subjects suffering from envenoming are currently able to access effective antivenoms [27], so the decision tree assessed the availability of effective antivenoms relative to the current standard of care of no availability in the decision node. As previously mentioned, other harmful snakes contribute to the overall burden from snakebites [10]–[14]. The distinction between carpet viper and other snakebites is made on the basis of the 20WBCT in the treatment arm of the model. Evidence of incoagulable blood would trigger the administration of mono-specific antivenom that neutralizes carpet viper venom only, whereas lack of evidence of incoagulable blood would trigger the administration of a polyspecific antivenom that neutralizes venoms from multiple snakes, including the carpet viper.

In the first chance node, the model included EARs associated with antivenom administration, which are more likely to occur with polyspecific rather than the monospecific antivenom [21], [22]. Symptoms of EAR include vomiting, urticaria, angioedema, itching, bronchospasm, laryngospasm and in severe cases anaphylactic shock developing rapidly within minutes of antivenom administration [21], and death in about 0.9% of cases [21], [22]. Survivors of snakebite may recover fully or remain with significant disability (e.g. limb amputation) that is factored in the model. Treatment outcomes were converted into DALYs on the basis of local life expectancy. No funding was needed for the study. All analyses were conducted using the Tree Age Pro Suite Healthcare 2014 software.

### Model Inputs

#### Antivenom effectiveness and Early Adverse Reactions (EAR) data

The probability of mortality from carpet viper and other snakebites was previously reported at 16% and 5%, respectively [5], [6], [14], [15]. Results from a systematic review and meta-analysis suggest that antivenom has a 75% effectiveness in reducing the risk of mortality from carpet viper bites [20]. As no placebo-controlled randomized controlled trials (RCT) have been conducted for antivenoms, the meta-analysis included solely observational studies from Chad, Ghana and Nigeria [6], [30], [31], [32]. As studies employing polyspecific antivenoms were included in the meta-analysis, it was assumed that the 75% reduction in the risk of mortality would also apply to non-carpet viper envenoming. This assumption was later relaxed in a scenario analysis. Thus, the absolute reduction in the risk of mortality associated with the provision of carpet viper antivenom was 12% (16% times the risk reduction of 75%) and assumed to be 3.75% (5% times the risk reduction of 75%) for antivenoms active against non-carpet viper poisoning.

The risk for EAR was obtained from a double-blind RCT in Nigeria that compared efficacy and safety of monospecific (EchiTab IgG) and polyspecific (EchiTab IgG Plus ICP) antivenoms [21], [25], [26]. The risk for disability (limb amputation) among survivors was obtained from studies in Nigeria and from sub-Saharan Africa [3], [14], [16]–[19]. Antivenom therapy was assumed not to reduce the risk of amputation in base-case analysis however effectiveness of 75% was applied during sensitivity analyses. In base-case analysis envenoming was assumed to occur at 26 years of age, the mean age of snakebite patients found in two large case series [21], [30]. In Nigeria, as at 2012, the remaining local life expectancy at that age is estimated to be 41 years [33]. Envenoming was also assumed to occur among victims in the age groups of 10–14 years and 40–44 years with respective remaining life expectancies of 53 years and 30 years applied in sensitivity analysis [33]. A discount rate of 3% annually was applied on the health outcome, yielding a discounted DALY estimate associated with early mortality of 23.41 [34], [35]. The probability of amputation among surviving patients was modeled at 3% [3] and the associated amputation-related disability weight was 0.102 [36], which yielded a discounted DALY estimate of 2.39.

#### Cost data

The cost of the full antivenom treatment regimen was modeled as US$125 [27]. The cost of care, initial and subsequent 20WBCT (10 times in 7 days for carpet viper and only once initially for diagnosis of non-carpet viper envenoming), transportation to-and-from hospital and feeding for 7 days, shipping and freezing of antivenom, management of EAR, supportive care - analgesics, blood transfusion, consumables, drugs, fluid rehydration, supportive care and wound care was obtained from series of envenomed patients admitted to Kaltungo General Hospital, Kaltungo, Gombe state, in northeastern Nigeria, see S1 Text and Table 1 [21], [30]. All costs were expressed in Nigerian Naira and converted to US$ using a current exchange rate of N160 = US$1. As snakebite is an acute condition and costs occur during a short span of time (two to ten days), costs were not discounted to adjust for time elapsed between expenditure and outcome during ICER calculations [34], [35].

**Table 1:** Base-case parameters and respective discounted ICER results for low and high values.

Parameter	Base-case value	Low*	High*	Low ICER value ($/DALY)	High ICER value ($/DALY)	Comments and Reference Source
Life expectancy at mean age of bite (26 years), or 25–29 years age group	41 years	30years #	53years∧	118.99#	88.43∧	[21], [30], [33]
Carpet viper causing envenoming (remainder being from non-carpet viper)	66%	0.0%	96%	254.18	78.25	[1], [6], [18]
Natural (untreated) mortality following carpet viper envenoming	16%	10%	30%	148.83	56.23	[1], [6]
Natural (untreated) mortality following non-carpet viper envenoming	5%	0%	10%	116.10	87.23	[5], [10], [11], [12], [14], [15]
Antivenom effectiveness to prevent death from carpet viper envenoming, Estimate[95%CI] (also applied to non-carpet viper envenoming)	75%	55%	86%	137.02	86.61	[20]
Risk of EAR – monospecific antivenom	19%	5%	30%	98.52	100.49	[21]
Risk of EAR – polyspecific antivenom	26%	5%	30%	98.77	99.78	[21]
Additional risk of mortality from EAR	1%	0.0%	2.5%	97.30	103.30	[22]
Risk of disability from amputation	3%	0.0%	5%	99.31	99.82	[3], [14], [19]
Disability weighting for finger/toe (0.102) and foot/hand (0.3) amputations, dw	0.102	0.00	0.300	99.31	100.21	[29], [36]
End-user cost of antivenom -monospecific [EchiTab]dose (1 vial) -polyspecific [EchiTab-Plus ICP] dose (3 vials)	$125	$50.0	$650	63.84	350.06	[27]
Cost of transportation to-from & feeding in Hospital for 7 days	$43.75 †	$25	$100.0	90.67	126.45	(a) Hospital patients and relations responses
Cost of supportive care (analgesia, blood transfusion, iv fluids, surgery, etc)	$18.75	-	-			(b) Hospital charges and formulary
Cost of testing for type of snake and adequacy of antivenom therapy (20WBCT), four times on day 1 and then daily for 6 days (10 tests)	$3.125	-	-			(b) Hospital charges
Cost of managing or premedicating EAR (adrenaline, antihistamines, syringes, etc)	$1.875	-	-			(b) Hospital charges and formulary
Cost of transporting and freezing antivenom	$18.75	-	-			Approximate estimate

*High and low ranges used in one-way sensitivity analyses; # life expectancy at age group 40–44 years ( = 30 years) with its corresponding ICER; ∧life expectancy at age group 10–14 years ( = 53 years) with its corresponding ICER; (a) interview responses/chart review of patients part published as reference [30]; (b) Hospital  =  Kaltungo General Hospital, Kaltungo, Gombe state, Nigeria.

† The sum of $43.75 was for feeding while in hospital at $3.125 per day for 7days ( = $21.875) and transportation to-and-from hospital ($21.875).

#### Sensitivity analysis

One-way sensitivity analysis and extreme value analyses were performed to test robustness and identify the most important variables influencing cost-effectiveness of an antivenom programme. Each base-case model input was varied independently according to the upper and lower limits from the relevant literature, and according to particular scenarios (see Table 1, low and high range).

## Results

**Figure 2:**
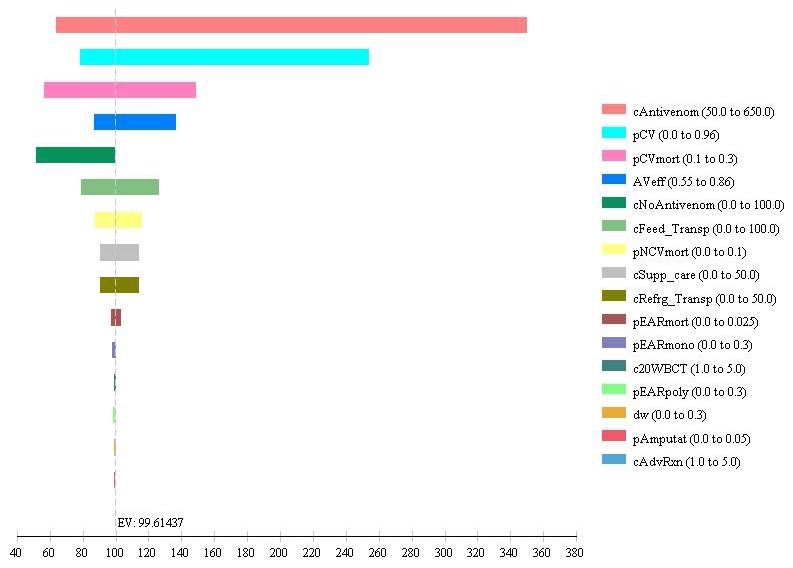
Tornado diagram assessing the impact of changes in envenoming/antivenom and cost parameters on the incremental cost-effectiveness ratio (ICER) per DALY for antivenom use in Nigeria. **Diagram Parameter Definitions**: c20WBCTest  =  cost of 20 minutes Whole Blood Clotting Test on 10 occassions over 7 days at diagnoses and monitoring; cAntivenom  =  Cost of Antivenom; cFeed_Transp  =  Cost of transporation and stay in Hospital for 7 days; cRefrg_Transp  =  Cost of shipping and refrigeration; cNoAntivenom  =  Cost of management without effective antivenoms either traditional/herbal care or other alternatives; cSupp_care  =  Cost of supportive care. All costs are in Nigerian Naira. antivenomeff  =  Effectiveness of antivenom to prevent death; pEARmono  =  probability of early adverse reactions with monospecific antivenom; pEARpoly  =  probability of early adverse reactionswith polyspecific antivenom; pEARmort  =  probability of dying following effective antivenom and early adverse reactions; pCVmort  =  probability of dying following carpet viper envenoming; pNCVmort  =  probability of dying following non-carpet viper envenoming; pCV  =  proportion of envenoming due to carpet viper; pDisabl =  probability of disability;

The average per patient cost for the full course of treatment, including complete testing, provision of antivenom, feeding and transportation to hospital, and supportive care was US$214.375. The cost of managing EAR averaged $1.875 per patient, resulting in a total average cost of antivenom of $216.25. The average decrease in the risk of mortality was 9.2%. Dividing the average antivenom cost by the absolute decrease in the risk of mortality yields a cost/Death averted of $2330.16. The average number of DALYs averted due to antivenom therapy was 40.88, thus, yielding a cost/DALY averted of $56.88 undiscounted. While the discounted average number of DALYs averted was 23.41, thus, yielding a cost/DALY averted of $99.61. The model results proved robust to variation of model parameters in one-way sensitivity analyses, see results in Table 1 and Fig. 2.

The projected cost-effectiveness was most sensitive to costs of antivenom, type of snake causing envenoming, costs of caring for envenoming without effective antivenom, efficacy of antivenom in reducing mortality and to natural (untreated) mortality following envenoming by carpet viper and non-carpet viper snakes (Fig. 2). Results were insensitive to prevalence of antivenom related EAR or the cost of caring for EAR (Fig. 2). Furthermore, results from scenario analysis in which the effectiveness of the polyspecific antivenom against non-carpet viper envenoming is reduced from 75% to 0% yield a cost/life saved of $2709.76 and a cost/DALY averted of $116.10. Antivenom cost is also varied from 80% ($100), 150% ($187.5) to 200% ($250) of the base-case price yielding cost/DALY averted of $87.69, $129.43 and $159.24 respectively. Applying a conservative reduction of 40% on risk of EAR due to adrenaline premedication [23], [24] yielded a cost/DALY averted of $98.67.

Utilizing probability of blindness of about 0.01% with a disability weight of 0.552 yielded an ICER of $99.32/DALY averted while utilizing a probability of Post-traumatic Stress Disorder (20%) with a disability weight of 0.105 yielded an ICER of $101.44 [14], [19], [36]. Similarly, antivenom was assumed to be ineffective on disability in the base-case analysis but applying 75% effectiveness on it yielded $97.20/DALY averted.

## Discussion

Economic modeling has been helpful in determining the most cost-effective and optimum way of managing conditions prevalent in resource constrained settings such as poisoning [37]. This is the first comprehensive study to assess the cost-effectiveness of making antivenom available to providers in the Nigerian setting. We find that the ICER associated with making antivenom available for patients presenting with snakebite envenoming in Nigeria was $2330.16 per death averted, $99.61 per DALY averted (discounted) and $56.88 per DALY averted (undiscounted). The results suggest that antivenoms for snakebite are highly cost-effective in Nigeria, as our estimates fall well below the commonly accepted, per capita income-based cost-effectiveness thresholds, which in the case of Nigeria is reported at $1555 [38], [39]. Our estimate of the cost per DALY averted also falls well below the gross domestic product (GDP) per capita of 16 countries in West Africa [38], [39] but it would be useful to formally replicate our analysis in other settings. The proportion of snakebites that are due to the carpet viper varies across West Africa; however, results from our sensitivity analysis suggest that it remains highly cost-effective to provide patients with access to antivenom even if the proportion of carpet viper bites is as low as 0%. A similar cost pattern would also be observed outside Africa in other developing country tropical settings where the cost of hospital stays and human resources are relatively low e.g., India. Our results are mainly driven by the availability of a simple, low-cost, sensitive, specific and reliable bed-side test for diagnosing and monitoring adequacy of antivenom therapy following carpet viper envenoming (the 20WBCT) combined with an effective and relatively inexpensive antivenom. Where such a discriminatory test is unavailable and several types of snakes could potentially cause different clinical manifestations of envenoming, the ICERs will likely be higher especially if more expensive tests are utilized e.g., venom antigen or molecular based tests. On the other hand, about 40% of victims present with the killed snake for identification and subsequent selection of the appropriate antivenom [21], which in these cases makes the discriminatory test redundant.

With limited healthcare resources and competing priorities, it may be useful to compare our estimates to those reported in other therapeutic areas. The cost-effectiveness of first line antiretroviral therapy has been previously estimated at US$620 per year of life gained in Cote d′Ivoire [40], and is likely to be even higher for second line antiretroviral therapy where annual treatment costs alone have been estimated at US$1,037 in South Africa [41]. When the added benefit of prevention in discordant couples is included, the cost per life year gained for this subgroup may be as low as US$530 in South Africa [42]. As a biological agent the cost effectiveness of antivenoms also compares favorably with other public health interventions that are generally considered worthwhile investments to improve population health, such as vaccines. The cost/DALY averted in our study is lower than the estimates of $142–$150/DALY averted for rotavirus vaccines reported from studies in Kenya and Pakistan [43], [44]. However, our results are comparable to cost effectiveness studies of Human Papilloma Virus vaccine and Pneumococcal conjugate vaccine that reported respective ICERs of ≤$100/DALY averted in 59 and 68 of 72 GAVI-eligible countries including Nigeria [45], [46].

In our sensitivity analysis, we estimate a cost/DALY averted of $76.08 if the model is restricted to carpet viper bites only. This is higher compared to an earlier study, which reported the cost effectiveness of antivenom following carpet viper envenoming at $10 per DALY averted [29]. Although slight methodological differences exist between these two models with respect the remaining life expectancy of the envenomed population as well as the mortality rate associated with carpet viper envenoming, the most impactful difference was in the antivenom cost. This input was previously modeled at the government subsidized rate of $40, compared to actual retail price of $125 in our model. Reducing our antivenom cost input to the government subsidized rate reduces our ICER estimate to $46.52 and, thus, more in line with previous estimates. Although most patients are managed with a dose of antivenom, few cases require more than one dose. And varying antivenom cost up to twice the base case price confirms they remain cost effective with an ICER of $159.24/DALY averted.

The antivenom efficacy used in the decision tree was based on the systematic review and meta-analysis of studies conducted in Chad, Ghana and Nigeria (West Africa) [20]. The meta-analysis referred to 6 non-RCT studies to produce estimates in respect to geographically appropriate and effective carpet viper antivenoms. The estimate is very conservative as observations in the field and one of the included studies reported antivenom conferred protection against death of 87% reducing mortality to as low as 1.29% [30]; thus, the ICER estimates will be lower with very effective antivenoms. A double-blind RCT directly comparing antivenom to placebo would provide better effectiveness data but would prove ethically challenging to conduct.

The present study has several limitations. First and foremost, the effectiveness of carpet viper antivenom relative to no antivenom administration in our model was not based on evidence from randomized clinical trials, but rather, on evidence obtained in observational analyses. As mentioned previously, it would not be ethical to conduct such a trial with a non-treated control group. Furthermore, we apply the relative risk reduction estimated for antivenoms against carpet viper envenoming to the polyspecific antivenom for non-carpet viper envenoming in the base case, although we later relax this assumption in a scenario analysis. Also, we do not consider other potentially relevant disability outcomes that have been anecdotally reported among snakebite survivors such as malignant ulcers and pregnancy loss [18]. Analyses were restricted to better recognized sequelae such as blindness or post-traumatic stress disorder both of which left the ICER estimates unchanged [14], [16], [19]. In addition, in our model, antivenoms reduce the risk of mortality only, although it is plausible that they may also reduce the risk of disability related endpoints such as amputations. Furthermore, the antivenom was not given credit for faster improvement/resolution of non-fatal conditions. Finally, while we do account for the marginal cost associated with the transportation and storage of the antivenom in refrigeration units, we assume that appropriate storage facilities already exist at the local level through immunization services and that no additional capital investment would be required to adequately store the antivenom in the field.

### Conclusions

The results of our study suggest that making antivenoms available to treat snakebite is highly cost-effective in Nigeria. A substantial expected reduction in mortality and DALYs could be achieved at a relatively modest upfront cost, thus, expanding access to antivenom to broader parts of the Nigerian population should be a priority consideration for future investments in healthcare.

## Supporting Information

S1 Text **Supporting information on the estimation of specific cost inputs.**
(DOC)
