# Supplementary material for: Cost-effectiveness of Antivenoms for Snakebite Envenoming in Nigeria
Source: PLoS Negl Trop Dis. 2015 Jan 8;9(1):e3381. doi: 10.1371/journal.pntd.0003381 (PMC4287484; doi:10.1371/journal.pntd.0003381)
Supplement: S1 Text — Supporting information on the estimation of specific cost inputs. (DOC) [file pntd.0003381.s001.doc]

**Text S1: Supplemental Information on the Estimation of Specific Cost Inputs**

1. End-user cost of antivenom

This was derived from the actual price paid by Kaltungo General Hospital Nigeria (KGH) snakebite patients of Twenty thousand Nigerian Naira or N20,000 per antivenom dose (=$125 at exchange rate of N160 to US$1). It is also consistent with a report of mean antivenom cost price of $124 (range, $50-650) by Brown 2012 [1]. We used the reported range in the sensitivity analysis.

1. Cost of transportation to-from & feeding in Hospital for 7 days

Patients’ and relations’ interviews at KGH were used to derive estimates of average cost of transportation and feeding. The estimates were derived in the course of surveys conducted by our group from 2009 to 2012 on 170 and 109 patients in the same hospital [2,3]. The sum of N7,000 ($43.75) was for feeding and transportation. Estimated average cost of feeding was N500 ($3.125) per day for 7days or N3,500 (=$21.875) and transportation to-and-from hospital was N3,500 (=$21.875).

1. Cost of supportive care (analgesia, blood transfusion, iv fluids, surgery, etc)

The average cost of supportive care is N3,000 (=$18.75) as derived from KGH formulary, hospital charges, records and patient payments. The money is for analgesia (paracetamol, tramadol), blood transfusion, intravenous rehydration fluids and surgery especially debridement. Many patients may not require blood transfusion or surgery.

1. Cost of testing for type of snake and monitoring adequacy of antivenom therapy using the 20minutes Whole Blood Clotting Test (20WBCT)

The 20minutes Whole Blood Clotting Test (20WBCT) entails use of a 5ml syringe (N15), small clean dry test-tube (N25) and accessories (local sterilization with methylated spirit and tourniquet (N10), totaling N50 for a test. Thus, the cost of 10 tests for carpet viper envenoming is N500 ($3.125) and for one test for non-carpet viper envenoming is N50 ($0.3125). These were obtained from hospital charges for the items.

2mls of fresh blood is collected and stood in the test-tube for 20minutes. Initial and subsequent 20WBCT (10 times in 7 days for carpet viper envenoming, with 4 tests conducted six hourly on the first day until restoration of clotting is confirmed and then daily for the remaining 6 days). The model utilizes only one 20WBCT done initially for diagnosis of non-carpet viper envenoming; a clotting blood in the 20WBCT confirms non-carpet viper envenoming.

1. Cost of managing or pre-medicating against Early Adverse Reactions (EAR)

The estimated average price is N300 ($1.875) and entails use of injections of adrenaline, antihistamines (chlopheniramine or promethazine injections) and or steroids (hydrocortisone injection). It comprises costs of 5ml syringe (N15) and the rest for drugs. Typically only one or few of the drugs are used. These were obtained from the KGH charges and formulary.

1. Cost of transporting and freezing (cooling) antivenom

The cost of shipping from abroad where the antivenoms are manufactured, transportation within Nigeria and freezing of antivenom (including use of supplementary diesel power electric generators in addition to national power grid) is estimated at N3,000 ($18.75) from prior experience and expert opinion. But it was assumed that appropriate storage facilities already exist at the local level through immunization/drug services and that no additional capital investment would be required to adequately store the antivenom in the field.

References

1. Brown NI (2012). Consequences of neglect: analysis of the sub-Saharan African snake antivenom market and the global context. PLoS Negl Trop Dis. 6:e1670.
2. Habib AG, Abubakar SB (2011). Factors affecting snakebite mortality in north-eastern Nigeria. International Health. 3: 50-55
3. Dalhat MM, Muhammad H, Abubakar S, Iliasu G, Yola IM, Habib AG (2012). Determinants of high cost of care among victims of snake bite in Kaltungo, Gombe state, Nigeria, 2009. 17^th^ World Congress of International Society of Toxinology and 4^th^ Venom Week (USA), Honolulu, Hawaii, USA, July 2012. Abstract #236. Toxicon 2012 (2): 216-217
